# Mass Spectrometry-Based Proteomic Analysis of Porcine Reproductive and Respiratory Syndrome Virus NSP9 Protein with Host Proteins

**DOI:** 10.3390/ani15243520

**Published:** 2025-12-05

**Authors:** Wei Wen, Yuhang Liu, Wenqiang Wang, Zhenbang Zhu, Xiangdong Li

**Affiliations:** 1 Jiangsu Co-Innovation Center for Prevention and Control of Important Animal Infectious Diseases and Zoonoses, College of Veterinary Medicine, Yangzhou University, Yangzhou 225009, China; 2 Joint International Research Laboratory of Agriculture and Agri-Product Safety, Ministry of Education of China, Yangzhou University, Yangzhou 225009, China

**Keywords:** PRRSV-NSP9, LC-MS/MS, PPI, CDK1

## Abstract

Porcine reproductive and respiratory syndrome virus (PRRSV) nonstructural protein 9 (NSP9), the viral RNA-dependent RNA polymerase (RdRp), is essential for viral replication but its comprehensive host interactome remains uncharacterized. This study employed co-immunoprecipitation coupled with liquid chromatography-tandem mass spectrometry (LC-MS/MS) to systematically identify NSP9-associated host proteins. We identified 222 high-confidence host interactors, with Gene Ontology and KEGG pathway analyses revealing significant enrichment in RNA/DNA-binding proteins, ubiquitin-proteasome pathways, metabolic regulators (amino acid/lipid biosynthesis), endoplasmic reticulum processing, and cell cycle components. Protein-protein interaction network analysis further delineated six functional modules involved in RNA processing, vesicular transport, and innate immunity. Crucially, validation studies confirmed direct binding between NSP9 and key candidates (CAPZ1, PSMA3, CDK1, USP48). Functional assessment demonstrated that CDK1 overexpression significantly inhibited PRRSV replication, implicating CDK1 as a host restriction factor. These findings collectively unveil the multifaceted role of NSP9 in subverting host machinery while identifying novel host defense mechanisms and potential targets for antiviral development against PRRSV.

## Simple Summary

This study maps how a key protein (NSP9) from the porcine reproductive and respiratory syndrome virus (PRRSV), which causes major losses in pig farming, hijacks host cell machinery to replicate. Using advanced proteomics, we identified 222 host proteins that interact with NSP9. Functional analysis revealed these proteins cluster in critical pathways including viral RNA synthesis, protein recycling systems (ubiquitin-proteasome), metabolic rewiring, and cell cycle control. We confirmed direct binding between NSP9 and four host proteins (CAPZ1, PSMA3, CDK1, USP48), and critically, demonstrated that overexpressing the cell cycle regulator CDK1 strongly inhibits PRRSV replication by reducing viral protein levels and infectious virus output. This identifies CDK1 as a novel host defense factor and reveals new targets for developing antiviral strategies against PRRSV.

## 1. Introduction

Porcine reproductive and respiratory syndrome virus (PRRSV) is the etiological agent of PRRS, a highly contagious disease that poses a severe threat to global swine production [1,2]. The syndrome is clinically characterized by reproductive failure in sows, including late-term abortions, stillbirths, and weak neonates, as well as respiratory distress in pigs of all ages [3]. Recognized as one of the most economically devastating pathogens in modern pig farming, PRRSV has inflicted profound annual losses on China’s pork industry, undermining both productivity and food security [4]. The virus evades host defenses through a combination of poor neutralizing antibody induction, attenuated cellular and innate immune responses, and generalized immunosuppression [5]. These mechanisms facilitate persistent infections and frequent co-infections with secondary pathogens, exacerbating disease severity [6,7,8]. While vaccination remains the primary prophylactic approach, the protective efficacy of existing PRRSV vaccines is inconsistent, highlighting an urgent need for novel therapeutic strategies [9]. A comprehensive understanding of PRRSV-host interactions, particularly at the protein-protein interface, is essential to elucidate viral pathogenesis and identify targets for intervention.

The PRRSV genome comprises a single-stranded, positive-sense RNA molecule approximately 15 kb in length, encoding at least 10 open reading frames (ORFs) [10]. ORF1a and ORF1b occupy the 5′-proximal region and are translated into polyproteins pp1a and pp1ab, which undergo extensive proteolytic processing to yield 14 nonstructural proteins (NSPs) [11]. Pp1a cleavage produces NSP1α, NSP1β, NSP2-NSP6, and two isoforms of NSP7 and NSP8, while pp1ab generates NSP9-NSP12-key components of the viral replication-transcription complex [12]. Recent studies have identified two additional NSPs, NSP2TF and NSP2N, derived from a ribosomal frameshift event within the replicase gene [13]. The structural proteome is encoded by ORF2a (GP2a), ORF3 (GP3), ORF4 (GP4), ORF5 (GP5), ORF2b (E), and ORF6 (M), with GP2a-GP5 forming N-glycosylated envelope proteins and E/M constituting non-glycosylated membrane-associated proteins [14]. The C-terminal ORF7 encodes the nucleocapsid (N) protein, which packages the viral genome into the virion core [15].

As obligate intracellular parasites, viruses rely extensively on host-pathogen protein interactions to hijack cellular processes and suppress antiviral defenses [16]. These interactions represent a dynamic: while host proteins attempt to restrict viral replication, viral effectors utilize or disrupt these defenses to promote propagation [17]. PRRSV exemplifies this paradigm, with prior studies using yeast two-hybrid and co-immunoprecipitation assays having mapped interactions between select viral proteins and host factors [18]. However, the interactome of NSP9, a multidomain replicase subunit essential for viral RNA synthesis, remains poorly characterized. Notably, no systematic, high-throughput proteomic analysis has been conducted to define the full spectrum of NSP9-binding partners, leaving a critical gap in our understanding of PRRSV replication mechanisms.

To address this knowledge gap, we employed an integrative proteomics approach combining co-immunoprecipitation (Co-IP) with liquid chromatography-tandem mass spectrometry (LC-MS/MS) to identify host proteins associated with NSP9 in transfected cells. Our analysis revealed 36 high-confidence interactors, which were used to construct a protein-protein interaction (PPI) network. Gene ontology enrichment revealed a significant overrepresentation of RNA/DNA-binding proteins, ribonucleoprotein complex subunits, and ATP-dependent helicases, consistent with NSP9’s role in viral RNA synthesis. 7 candidate interactors were selected for validation, of which four (CAPZ1, PSMA4, CDK1, USP48) exhibited binding to NSP9 in vitro. These findings not only expand the known repertoire of PRRSV-host interfaces but also nominate potential targets for broad-spectrum antiviral development.

## 2. Materials and Methods

### 2.1. Cells and Viruses

MARC-145 cells and HEK293T cells were maintained in Dulbecco’s Modified Eagle Medium (DMEM) (Gibco, C11995500BT, New York, NY, USA) supplemented with 10% heat-inactivated fetal bovine serum (Gibco, 16000044, New York, NY, USA). All cells were incubated at 37 °C/5% CO_2_. PRRSV was propagated in MARC-145 cells.

### 2.2. Antibodies and Reagents

Primary antibodies included mouse anti-HA (66006-2-Ig), rabbit anti-HA (51064-2-AP), mouse anti-Flag (66008-3-Ig), mouse anti-α-tubulin (6031-1-Ig), and HRP-conjugated goat anti-mouse IgG (SA00001-1) were purchased from Proteintech (Wuhan, China). The anti-PRRSV N monoclonal antibody was developed and preserved in our laboratory.

### 2.3. Plasmid Construction and Transfection

The PRRSV NSP9 gene was cloned via RT-PCR into pCAGGS-HA to generate HA-NSP9. Host genes (FAM134C, CAPZ1, PSMA4, PSMA3, CDK1, USP48, and TPM3) were amplified from MARC-145 cells and cloned into pcDNA3.1-3Flag. Plasmids were sequence-verified. HEK293T or MARC-145 cells at 70% confluency were transfected with Jetprime (Polyplus, 101000046, Illkirch, France) per the manufacturer’s protocol.

### 2.4. Western Blotting

Cells were lysed in buffer (5% SDS, 1% Triton X-100, 50 mM Tris-HCl, 150 mM NaCl), denatured in 5× loading buffer (100 °C, 5 min), separated by SDS-PAGE, and transferred to PVDF membranes. After blocking with 5% non-fat milk/0.1% Tween-20 (Solarbio, T8220, Beijing, China; RT, 1 h), membranes were incubated with primary antibodies (RT, 1 h) and HRP-conjugated secondaries (RT, 1 h). An extremely sensitive ECL Chemiluminescence Detection Kit (Thermo, Shanghai, China) was then used to detect the signals.

### 2.5. Co-Immunoprecipitation (Co-IP)

Cells were harvested and lysed in ice-cold lysis buffer (1.19% HEPES, 0.88% NaCl, 0.04% EDTA, 1% NP-40) supplemented with a protease inhibitor (bimake, B140115, Houston, TX, USA), followed by incubation on ice for 30 min. Cell lysates were first incubated overnight at 4 °C with anti-HA or anti-Flag monoclonal antibodies under continuous rotation. The samples were then mixed with Protein A + G Agarose (Beyotime Biotechnology, P2012, Wuhan, China) and incubated for 3 h at 4 °C. After extensive washing with cold lysis buffer (five times), the immunoprecipitated complexes were subjected to immunoblotting analysis.

### 2.6. LC–MS/MS Analysis

For LC-MS/MS analysis, immunoprecipitated complexes were resolved via SDS-PAGE, followed by silver staining to visualize differentially expressed protein bands between experimental and control groups. Selected bands were excised and subjected to LC-MS/MS by APTBIO (Shanghai, China) to identify NSP9-interacting proteins. The resulting peptides were concentrated and desalted on an EASY column (2 cm × 100 μm, 5 μm-C18), followed by online separation on an analytical RP column (75 μm × 150 mm, 3 μm C18) with a 60-min linear gradient. The gradient was set as follows: 4–50% B over 0–50 min, 50–100% B over 50–54 min, and 100% B over 54–60 min (Solvent A: 0.1% formic acid; Solvent B: 0.1% formic acid in 84% acetonitrile). Database searches were conducted with Mascot 2.2, and the resulting identifications were filtered by removing proteins found in negative controls and retaining only those with at least two unique peptides for subsequent analysis.

### 2.7. Bioinformatics

NSP9-interacting proteins were annotated for GO terms and KEGG pathways using DAVID. Enrichment significance was assessed by Fisher’s exact test. Protein interactions were mapped via STRING.

### 2.8. Statistical Analysis

Data are the mean ± SD. Group comparisons used two-tailed * *t*-tests (GraphPad Prism v7.0), with * *p* < 0.05 considered significant.

## 3. Results

### 3.1. Identification of Host Protein Interacting with PRRSV NSP9 by Liquid Chromatography Mass Spectrometry

**Figure 1:**
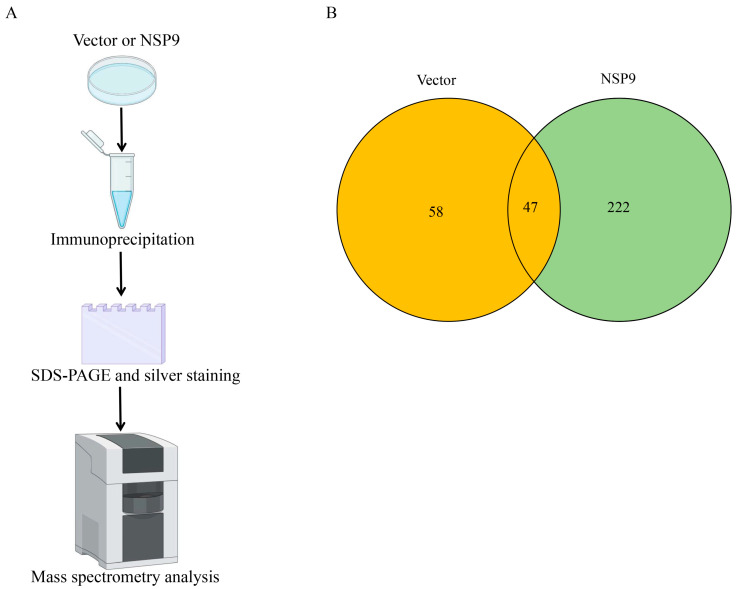
Cellular proteins interacting with PRRSV NSP9 were identified by co-immunoprecipitation (Co-IP). (**A**) Cells expressing HA-NSP9 for 30 h were subjected to Co-IP. Immunoblotting of lysates confirmed interactions were excised for bound protein identification via LC–MS/MS. (**B**) Venn diagram of proteins co-precipitating with PRRSV NSP9 versus vector control.

Porcine reproductive and respiratory syndrome virus (PRRSV) nonstructural protein 9 (NSP9) functions as the viral RNA-dependent RNA polymerase (RdRp), playing a central role in viral genome replication by catalyzing the synthesis of complementary RNA strands. Structural and biochemical evidence indicates that NSP9 interacts with other viral nonstructural proteins (NSPs) to form the replication-transcription complex (RTC), a multi-protein machinery essential for efficient viral RNA synthesis. However, the host cellular proteins that associate with NSP9 to facilitate viral replication remain poorly characterized. To systematically identify NSP9-interacting host factors, we conducted co-immunoprecipitation (Co-IP) assays coupled with liquid chromatography-mass spectrometry (LC-MS/MS). HEK-293T cells were transiently transfected with an HA-tagged NSP9 construct for 36 h, followed by lysis and immunoprecipitation using an anti-HA monoclonal antibody. To eliminate non-specific interactions, lysates from mock-transfected cells were processed in parallel as a negative control. The immunoprecipitated proteins were subjected to high-resolution LC-MS/MS analysis (Figure 1A). Our proteomic screening identified 269 putative host cellular proteins that co-precipitated with NSP9. To enhance the specificity of our findings, we subtracted 47 proteins detected in the mock control, yielding 222 high-confidence NSP9-associated host factors (Figure 1B and Supplementary Table S1).

### 3.2. Functional Characterization of NSP9-Interacting Proteins Reveals a Role in Post-Translational Modifications

**Figure 2:**
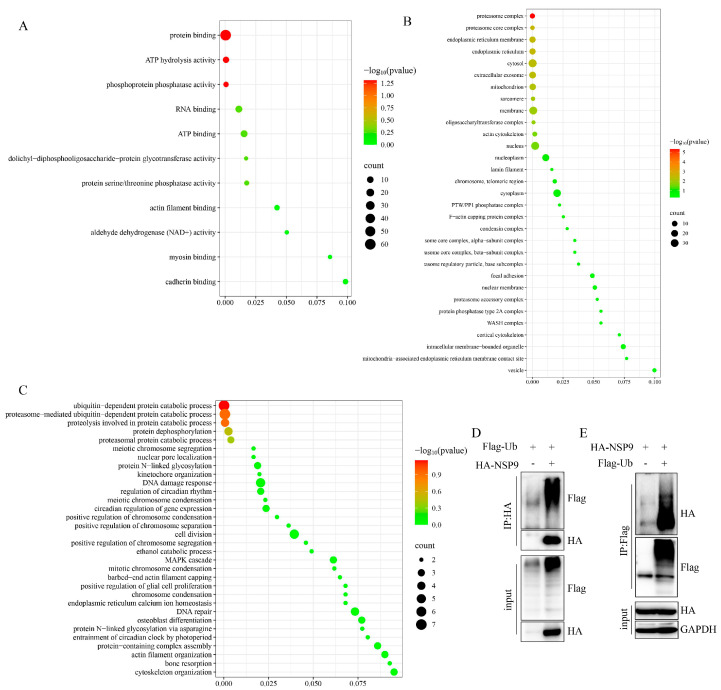
Functional annotation of PRRSV NSP9-interacting proteins. (**A**) Biological processes, (**B**) Cellular components, (**C**) Molecular functions. Top 20 enriched Gene Ontology (GO) terms are shown. The hyper-geometric test’s *p* value is shown by the color of the dot. The color spectrum includes red and green. Enrichment score is plotted on the abscissa. (**D**,**E**) Verifying the ubiquitination of NSP9 protein.

To elucidate the functional properties of NSP9-interacting proteins, we performed Gene Ontology (GO) enrichment analysis. All identified genes had corresponding GO annotations, which were classified into three major categories (Figure 2). Within biological processes, NSP9-associated proteins were predominantly enriched in the ubiquitin-proteasome pathway, proteolysis, protein dephosphorylation, and chromosome condensation (Figure 2A), suggesting their involvement in coordinated regulatory mechanisms. The cellular component analysis revealed that these proteins localized primarily to the proteasome complex, endoplasmic reticulum, and membrane-bounded organelles (Figure 2B), implicating their roles in specific subcellular structures. At the molecular function level, the most prominent activities included protein binding, ATP hydrolysis, and phosphoprotein phosphatase activity (Figure 2C). These findings strongly implicate protein post-translational modifications (PTMs), particularly ubiquitination, in NSP9 function. Consistent with this, co-immunoprecipitation (Co-IP) assays confirmed that NSP9 undergoes ubiquitination (Figure 2D,E).

### 3.3. KEGG Pathway Annotation of Host Proteins Interacting with PRRSV NSP9

**Figure 3:**
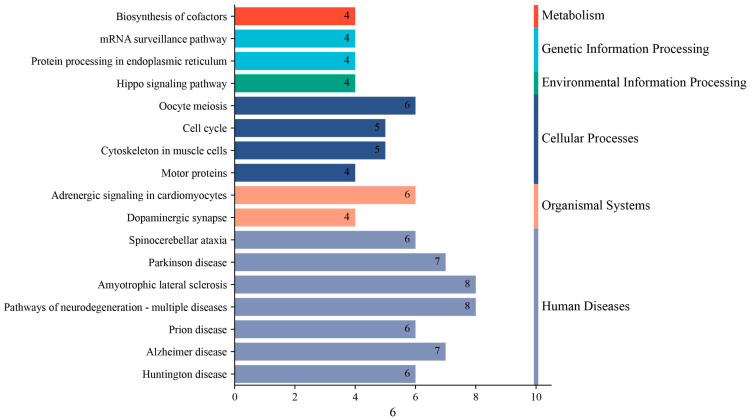
KEGG pathway enrichment analysis of NSP9-interacting proteins.

To elucidate the functional pathways associated with host proteins interacting with porcine reproductive and respiratory syndrome virus (PRRSV) nonstructural protein 9 (NSP9), we performed a comprehensive KEGG pathway enrichment analysis using stringent statistical criteria. The bioinformatics analysis identified 17 significantly enriched pathways (Figure 3), which were systematically categorized into three major functional clusters: (1) genetic information processing (including mRNA surveillance pathway and spliceosome), (2) cellular processes (particularly cell cycle regulation and cytoskeletal dynamics involving motor proteins), and (3) metabolic regulation (especially amino acid metabolism and lipid biosynthesis). Intriguingly, the most prominent enrichment was observed in metabolic pathways, suggesting NSP9 may preferentially target host metabolic machinery. Furthermore, the significant involvement of endoplasmic reticulum protein processing pathways implies potential viral manipulation of secretory pathways. These findings collectively provide novel insights into the molecular mechanisms by which NSP9 modulates host cellular processes to facilitate viral replication and pathogenesis.

### 3.4. Protein-Protein Interaction Network of NSP9-Associated Host Proteins

**Figure 4:**
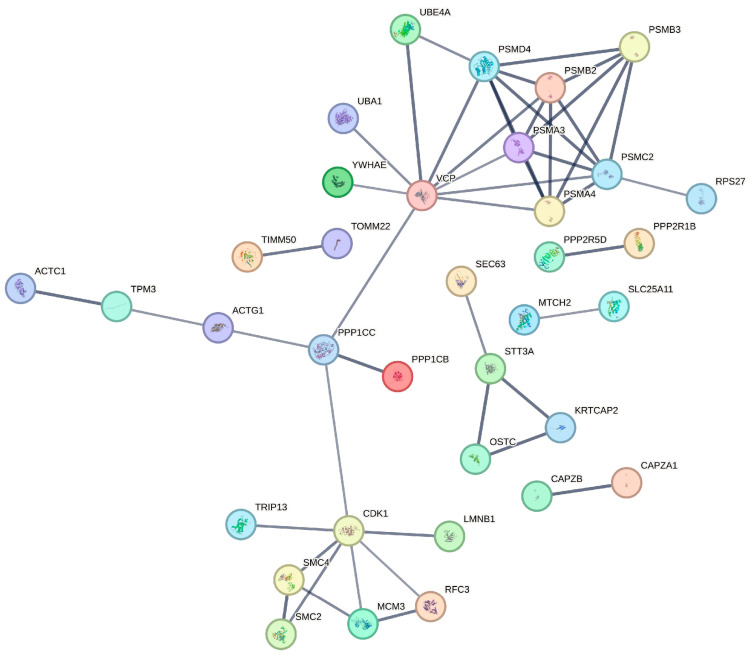
Using the STRING database, we generated the NSP9 protein-protein interaction (PPI) network and clustered interacting partners with a confidence value above 0.7, designating functional clusters by color.

To systematically investigate functional relationships among NSP9-interacting host proteins and identify potential multi-protein complexes, we constructed a protein-protein interaction (PPI) network using STRING database predictions with high-confidence interaction scores. The resulting network comprised 36 candidate proteins (Figure 4). Network topology analysis revealed a scale-free architecture with several highly connected hub proteins. We identified five distinct, tightly interconnected protein modules, which exhibited significantly enriched functional annotations. Notably, proteins within each cluster demonstrated strong co-expression patterns and functional coherence, suggesting their cooperative involvement in specific biological processes such as RNA processing, vesicular transport, and innate immune signaling pathways. This modular organization implies that NSP9 may target functional protein complexes rather than individual host factors during infection.

### 3.5. CDK1 Expression Inhibited PRRSV Replication

**Figure 5:**
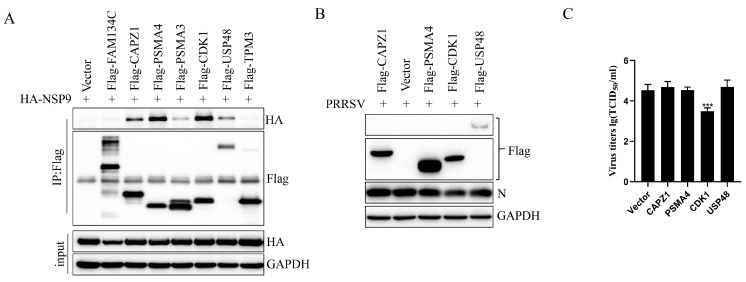
CDK1 inhibits PRRSV replication. (**A**) HEK293T cells were co-transfected with HA-tagged expression vectors for the selected host proteins and incubated for 24 h. Cell lysates were then immunoprecipitated using anti-HA antibodies, followed by immunoblotting with the corresponding primary antibodies. (**B**,**C**) MARC-145 cells were transfected with the indicated proteins for 24 h, followed by infection with PRRSV at an MOI of 0.1 for an additional 24 h. Western blot analysis was performed on the cell lysates, and the virus titer was assessed in the collected supernatant. Student’s *t*-test: *** *p* < 0.001 against control.

Based on the mass spectrometry analysis, we selected several top-ranked candidate proteins, including FAM134C, CAPZ1, PSMA4, PSMA3, CDK1, USP48, and TPM3, for further validation. Co-immunoprecipitation assays revealed that CAPZ1, PSMA3, CDK1, and USP48 could physically interact with NSP9 (Figure 5A), suggesting their potential involvement in the PRRSV life cycle. To explore the functional significance of these interactions, we overexpressed each of these proteins in susceptible cells and subsequently infected them with PRRSV for 24 h. Interestingly, overexpression of CDK1 resulted in a significant reduction in the expression levels of the viral N protein (Figure 5B), a key marker of PRRSV replication. Furthermore, viral titration assays demonstrated that CDK1 overexpression led to a notable decrease in viral titers (Figure 5C), indicating its inhibitory effect on PRRSV propagation. These findings suggest that CDK1 may serve as a host restriction factor that negatively regulates PRRSV replication. The observed interaction between CDK1 and NSP9 raises the possibility that CDK1 might interfere with the function of the viral replication and transcription complex, in which NSP9 plays a crucial role. Alternatively, CDK1 may modulate host cellular pathways that are essential for viral replication. Further studies are warranted to elucidate the precise molecular mechanisms underlying the antiviral activity of CDK1 against PRRSV. The identification of CDK1 as a potential host restriction factor provides new insights into the host-virus interactions during PRRSV infection and may contribute to the development of novel antiviral strategies. Moreover, the interactions between NSP9 and other host proteins, such as CAPZ1, PSMA3, and USP48, warrant further investigation to determine their roles in the PRRSV life cycle. Understanding these virus-host interactions will enhance our knowledge of PRRSV pathogenesis and may reveal additional targets for antiviral intervention.

## 4. Discussion

Understanding the intricate interplay between viral and host proteins is fundamental to deciphering PRRSV infection mechanisms and host responses. Central to this process is NSP9, the core RNA-dependent RNA polymerase (RdRp) within the viral replication and transcription complex (RTC) [19]. NSP9’s RdRp activity is indispensable for PRRSV replication, serving as the pivotal regulator of nucleotide selectivity and fidelity [20]. Furthermore, NSP9 functions as an emerging protein interaction nexus, engaging both viral and host partners to modulate replication efficiency and contributing significantly to the enhanced pathogenicity of HP-PRRSV strains through immunosuppressive activities [21,22]. Its high sequence conservation across PRRSV strains positions NSP9 as a prime target: for developing novel antiviral strategies and vaccines by disrupting its essential functions, and for creating sensitive, broadly reactive diagnostic assays [23]. Thus, NSP9 represents a critical molecular focal point for both fundamental virology and applied interventions against PRRSV [24]. This study delineates the first comprehensive host interactome of PRRSV NSP9, the viral RNA-dependent RNA polymerase, through an integrated immunoprecipitation and high-resolution mass spectrometry approach. The identification of 222 high confidence host binding partners reveals the multifaceted role of NSP9 in subverting cellular machinery while simultaneously exposing novel host defense mechanisms. The functional diversity of these interactors extends far beyond NSP9’s canonical role in viral RNA synthesis, positioning it as a central coordinator of extensive host pathogen crosstalk during infection. It is important to note that our Co-IP/MS screen was performed in HEK293T cells. While this system offers advantages for high-efficiency transfection and protein production, it lacks many of the specialized immune functions of primary porcine macrophages. Therefore, the protein interactions identified here should be considered as a preliminary map of potential interactors, and their physiological relevance must be confirmed in more biologically relevant systems.

A particularly significant finding is the pronounced enrichment of NSP9-associated proteins involved in post translational modification networks, especially ubiquitination, proteasomal degradation, and dephosphorylation pathways. This compellingly suggests that NSP9 exploits host PTM systems to regulate its own stability, enzymatic activity, or immune evasion capabilities, a strategy directly supported by our validation of NSP9 ubiquitination. Such manipulation aligns with established mechanisms employed by diverse RNA viruses including coronaviruses [25,26]. Equally notable is the robust enrichment of metabolic regulators governing amino acid metabolism and lipid biosynthesis, indicating that NSP9 actively reprograms host metabolism to redirect energy and biosynthetic precursors toward viral replication. The concurrent identification of endoplasmic reticulum protein processing components further implies that NSP9 may disrupt ER homeostasis or secretory pathways. associations with cytoskeletal proteins like CAPZ1 and TPM3 suggest potential roles for NSP9 in intracellular trafficking or virion assembly, while interactions with proteasomal subunits and deubiquitinases reinforce the critical importance of post translational modification networks in the viral life cycle.

Protein interaction network analysis revealed that NSP9 engages with organized functional modules rather than isolated host factors. These tightly interconnected complexes span RNA processing machinery, proteasomal components, cytoskeletal regulators, and cell cycle controllers, suggesting NSP9 acts as a molecular scaffold that simultaneously adopts multiple cellular subsystems. Within this framework, the validation of CDK1 as both a direct NSP9 interactor and a potent inhibitor of PRRSV replication represents a pivotal discovery. CDK1 overexpression significantly suppressed viral N protein expression and reduced viral titers, establishing its role as a host restriction factor. While CDK1 primarily regulates cell cycle progression, its antiviral function may arise through direct interference with the replication transcription complex via NSP9 binding, induction of cell cycle states incompatible with efficient replication, or modulation of innate immune signaling pathways. This finding resonates with emerging evidence that cell cycle regulators restrict diverse viruses and highlights CDK1 as a compelling target for host-directed antiviral strategies.

Collectively, this work illuminates the complex interface between PRRSV NSP9 and the host proteome, revealing not only sophisticated viral hijacking mechanisms but also intrinsic host defenses exemplified by CDK1. These findings substantially advance our understanding of PRRSV pathogenesis and provide a rich repository of targets for rational antiviral development. Future investigations should dissect the precise molecular consequences of key interactions, particularly how CDK1 impedes replication and whether NSP9-directed metabolic rewiring is essential for infectivity. Targeting these newly identified host dependencies or viral interfaces holds significant promise for developing urgently needed countermeasures against this economically devastating pathogen.

## 5. Conclusions

Based on the comprehensive proteomic and functional analyses presented in this study, we conclude that PRRSV NSP9 serves as a central hub for extensive virus-host interactions, engaging 222 high-confidence host proteins. The interactome of NSP9 spans multiple cellular processes, including RNA processing, ubiquitin-proteasome regulation, metabolic reprogramming, and cell cycle control, underscoring its multifunctional role beyond canonical RNA synthesis. Crucially, we identified CDK1 as a novel host restriction factor that directly binds NSP9 and significantly inhibits PRRSV replication. These findings not only deepen our understanding of how PRRSV subverts host machinery but also unveil new layers of host defense mechanisms. The identification of key host interactors, particularly CDK1, provides promising targets for the development of host-directed antiviral strategies against PRRSV, offering potential avenues to mitigate the impact of this economically devastating pathogen.

## Data Availability

The original contributions presented in this study are included in the article. Further inquiries can be directed to the corresponding author.

## Supplementary Materials

The following supporting information can be downloaded at: https://www.mdpi.com/article/10.3390/ani15243520/s1. Table S1: NSP9 interacting protein.
